# Pulse Oximetry for the Detection of Obstructive Sleep Apnea Syndrome: Can the Memory Capacity of Oxygen Saturation Influence Their Diagnostic Accuracy?

**DOI:** 10.1155/2011/427028

**Published:** 2011-10-19

**Authors:** Carlos A. Nigro, Eduardo Dibur, Edgardo Rhodius

**Affiliations:** ^1^Sleep Laboratory, Hospital Alemán, Pedro Goyena 620, 3 B, CP 1424 Buenos Aires, Argentina; ^2^Sleep Laboratory, Pneumonology Unit, Hospital Alemán, Puyrredon 1640, CP 1418 Buenos Aires, Argentina

## Abstract

*Objective*. To assess the diagnostic ability of WristOx 3100 using its three different recording settings in patients with suspected obstructive sleep apnea syndrome (OSAS). *Methods*. All participants (135) performed the oximetry (three oximeters WristOx 3100) and polysomnography (PSG) simultaneously in the sleep laboratory. Both recordings were interpreted blindly. Each oximeter was set to one of three different recording settings (memory capabilities 0.25, 0.5, and 1 Hz). The software (nVision 5.1) calculated the adjusted O_2_ desaturation index-mean number of O_2_ desaturation per hour of analyzed recording ≥2, 3, and 4% (ADI2, 3, and 4). The ADI2, 3, and 4 cutoff points that better discriminated between subjects with or without OSAS arose from the receiver-operator characteristics (ROCs) curve analysis. OSAS was defined as a respiratory disturbance index (RDI) ≥ 5. *Results*. 101 patients were included (77 men, mean age 52, median RDI 22.6, median BMI 27.4 kg/m^2^). The area under the ROCs curves (AUC-ROCs) of ADI2, 3, and 4 with different data storage rates were similar (AUC-ROCs with data storage rates of 0.25/0.5/1 Hz: ADI2: 0.958/0.948/0.965, ADI3: 0.961/0.95/0.966, and ADI4: 0.957/0.949/0.963, *P* NS). *Conclusions*. The ability of WristOx 3100 to detect patients with OSAS was not affected by the data storage rate of the oxygen saturation signal. Both memory capacity of 0.25, 0.5, or 1 Hz showed a similar performance for the diagnosis of OSAS.

## 1. Introduction

The obstructive sleep apnea syndrome (OSAS) is characterized by partial or total closure of the pharynx during sleep, which produces irregular episodes of hypopnea or apnea associated with oxygen desaturation of variable magnitude. The degree of oxygen desaturation can depend on a number of factors including baseline level of oxygen saturation, lung volume, sleep stage, type and duration of respiratory events, location of the sensor, and technological differences between the oximeters [1–5]. Other parameters that can influence the morphology of the SO_2_ signal and the quantification of such data are the acquisition parameters including the averaging time and data storage rate of the SO_2_ signal [6–9]. Averaging time refers to the time window used by the device in order to produce a moving average of the data stream that works as a signal filter, smoothing out short term fluctuations. Different averaging times can produce different results with the same oximeter [7, 10], and also the same averaging time can lead to different results using different oximeters [5]. Three reports have showed that when the averaging time and the data storage rate of the SO_2_ are shorter, the oxygen desaturation index (ODI) and the respiratory disturbance index (RDI) are generally higher and vice versa [10–12]. However, no study has evaluated yet whether the observed differences in the ODI with different acquisition parameters could affect the performance of pulse oximetry in terms of sensitivity and specificity for diagnosing OSAS. The Nonin WristOx 3100 is a small, lightweight pulse oximeter designed to be worn comfortably on the patient's wrist. This type of device is ideal for use in the patient's home. This can be set to specific data storage rates for oxygen saturation signal (1, 0.5, 0.25 Hz), allowing a greater or lesser capacity for data storage in the device memory. We have recently validated the WristOx 3100 oximeter with polysomnography to diagnose OSAS [13]. At that time, the oximeter was set to data storage rate of SO_2_ of 0.5 Hz. Therefore, the objective of this study was to assess the diagnostic ability of WristOx 3100 using its three different recording settings in patients with suspected OSAS.

## 2. Methods

### 2.1. Patient Selection

A prospective clinical study was performed in 138 consecutive patients referred to the Hospital Alemán Sleep Laboratory for investigation of possible OSAS. The recruitment period extended from July 2009 to July 2010. The patients were derived by different specialities (Otorhinolaryngology, Pneumonology, Internal Medicine, Cardiology, and Neurology). The selection criteria were the following.

- *Inclusion criteria.*
  1. OSAS-suspected patients of both sexes (snoring with/without other symptoms such as apneas referred by someone and/or somnolence).
  2. Age equal to or over 18 years old.
  3. Patient's consent for taking part in the study.
- *Exclusion criteria.*
  1. Use of oxygen, CPAP, or some modality of noninvasive mechanical respiratory assistance during PSG.
  2. Age under 18 years old.
  3. Patients suspected of having congestive heart failure, neuromuscular disease, insomnia, parasomnias, periodic limb movement disorder, circadian rhythm disorders, or narcolepsy.
- *Elimination criteria.*
  1. Polysomnographies with artifacts in EEG or respiratory channels (airflow, toracoabdominal movements, and SO_2_) that did not allow the reading of the sleep stages or the respiratory events.
  2. Total sleep time less than 180 min in the PSG.
  3. Oximetry with artifacts by disconnections or finger probe displacement.
  4. A difference greater than or equal to 5 minutes between the analyzed times of the oximeters.

All patients had a PSG and an oximetry (Nonin WristOx 3100) performed simultaneously in the Sleep Laboratory. An institutional review board approved the study protocol.

### 2.2. Measurements

#### 2.2.1. Polysomnography

Prior to the polysomnography, patients completed the Berlin [14] questionnaire and a clinical history. All the subjects underwent PSG with a computerized polysomnographic system (NEUROTRACE or MINI-PC; Akonic, Bs. As., Argentina), including electroencephalogram (F4/A2, C4/A2, and O2/A2), bilateral electrooculogram, submental electromyogram, bilateral leg electromyogram, and electrocardiogram. Airflow was measured by nasal cannula; respiratory effort was assessed by thoracic and abdominal piezoelectric belts and SO_2_ was recorded using a finger probe (Nonin, OEM III). The polysomnographies were registered from 10.30–11.30 PM to 05-06 AM. On the day of the study, the patients were given the following instructions: (1) to avoid napping and not to drink alcohol or beverages with caffeine (coffee, tea, and cola drinks); (2) to continue the usual medication; (3) to eat supper between 8.30 and 9.30 PM; (4) to report to the sleep lab between 10.30 and 11.30 PM.

**PSG Analysis**

PSG reading was performed manually by two widely experienced medical staff members who were blind to the operator that analyzed the oximetry. The sleep stages were analyzed in 30 s epochs according to international criteria [15]. The arousals were identified following the American Sleep Disorder Association recommendations [16]. The analysis of apneas, hypopneas, and respiratory effort related arousals (RERAs) were in agreement with the international criteria [17, 18]. The following definitions were used.

- Respiratory disturbance index (RDI): number of apneas plus hypopneas plus RERAs per hour of sleep [19].
- OSAS was defined as an RDI ≥ 5.
- Severity of OSAS: mild = RDI ≥ 5–< 15; moderate = RDI ≥ 15–< 30; severe RDI ≥ 30 [20].

#### 2.2.2. Pulse Oximetry

The Nonin WristOx 3100 (Nonin, Plymouth, Minn, USA) was used to compare its diagnosis accuracy with respect to PSG. Each patient had three sensors placed on the distal aspect of three fingers, which were connected to the three separate oximeters. Each oximeter was set to one of three different recording settings (0.25, 0.5, and 1 Hz data storage rate). The response time of the SO_2_ signal is based on an exponential averaging every 4 or 8 beats depending on the heart rate. This recording parameter cannot be modified by the user. The nail polish was removed to avoid interferences in the reading of SO_2_.

**Analysis of SO_2_**

A blind independent observer of the PSG results performed the automatic analysis with the nVision 5.1 software (Nonin, Plymouth, Minn, USA). The software performed an automatic SO_2_ data exclusion of areas with artifacts. Besides, the observer that analyzed the oximetry could manually exclude from analysis those areas that showed a clear disconnection of the finger probe (SO_2_ or cardiac frequency signal loss). The automatically and manually excluded data constituted the time of artifacts. Thus, the analyzed time was the total recording time minus the time of artifacts. The nVision program calculated the following variables.

- Basal SO_2_ is the average of the SO_2_ readings that are not included in any desaturation event.
- O_2_ desaturation event (D): a decrease in SO_2_ from basal SO_2_ ≥ 2, 3, and 4% (D2, D3, and D4, respectively) lasting at least 12 s.
- Adjusted O_2_ desaturation indexes (ADIs): mean number of O_2_ desaturations per hour of analyzed recording ≥ 2, 3, and 4% (ADI2, 3, and 4).

The ADI2, 3, and 4 cutoff points that better discriminated between subjects with or without OSAS arose from the receiver operating characteristic (ROC) curve analysis.

### 2.3. Statistical Analysis

To assess if the study variables had a normal distribution, we performed a frequency histogram and used the Kolmogorov-Smirnov test. Thus, when the distribution was normal, the mean and standard deviation were reported. Instead, the median and the 25–75% percentiles were used if the distribution was not normal. The chi-square test was used to evaluate significant differences between patients with mild, moderate, and severe sleep apnea. A repeated-measures analysis of variance was performed to determine whether there were differences between analyzed times of the oximetry and the ADIs at the three data storage rates. The diagnostic accuracy of oximetry using different cutoff points of RDI was evaluated by the receiver operating characteristics (ROCs) curve. Similarly, we compared the area under the ROC curve of the best ADI2, 3, and 4 cutoff points. Sensitivity and specificity, positive and negative likelihood ratio (LR+, LR−) were assessed for the different criteria of positive oximetry. The Mann Whitney or the unpaired *t*-tests were used to compare the differences between false negative (FN) versus true positive (TP) and false positive (FP) versus true negative (TN) cases depending on the variable distribution. The statistical analysis was made with a commercially available software programme (MedCalc Software, Version 11.3, Mariakerke, Belgium and Meta-DiSc Version 1.4, Universidad Complutense, Madrid, Spain). 

## 3. Results

Out of the 138 patients who were invited into the study, 135 gave informed consent. Mechanical equipment malfunctioning or artifacts by disconnections or finger probe displacement in one or more oximeters occurred in 26 patients, technologist error (failure to properly download memory data, errors in the configuration of the data storage rate of oximeter) was noted in 4 cases and other 4 cases had a total sleep time less than 180 min. Thus, 101 subjects provided acceptable data for comparing the influence of recording settings on the performance of the oximeter. 

### 3.1. Subjects Characteristics

The patient characteristics are shown in Table 1. Men represented 76% of the study sample. The prevalence of OSAS ranged from 68% to 83%, depending on the cutoff point adopted to define OSAS. 57% of the study sample had mild to moderate OSAS and 43% of subjects had severe sleep apnea. 6 patients with an AHI < 5 in the PSG were classified as mild OSAS when the RERA was included in the analysis. 17 patients categorized as mild or moderate OSAS by AHI were reclassified as moderate and severe OSAS by the addition of the RERA (AHI 12.6 ± 1.7 versus RDI 18.3 ± 1.7; AHI 25.5 ± 2.7 versus RDI 33.3 ± 2.6). The analyzed time of the oximetry for each data storage rate was similar (1 s: 421.1 ± 22.3; 2 s: 421.2 ± 22.3; 4 s: 421.1 ± 22.2, *P* 0.6).

### 3.2. Agreement between Oximetry and Polysomnography

**Figure 1:**
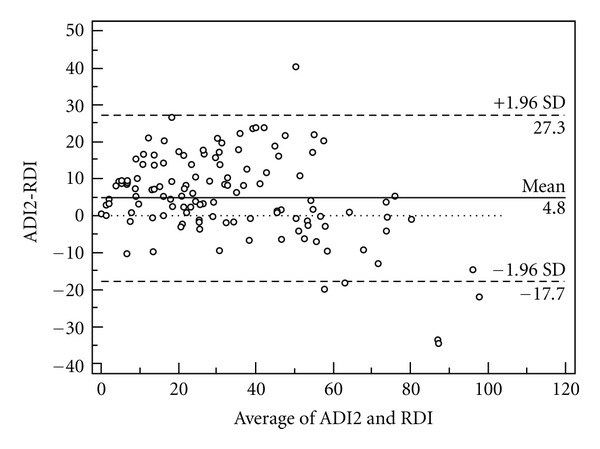
Bland-Altman plot of ADI2 and the respiratory disturbance index (RDI) from PSG (data storage rate 0.5 Hz).

**Figure 2:**
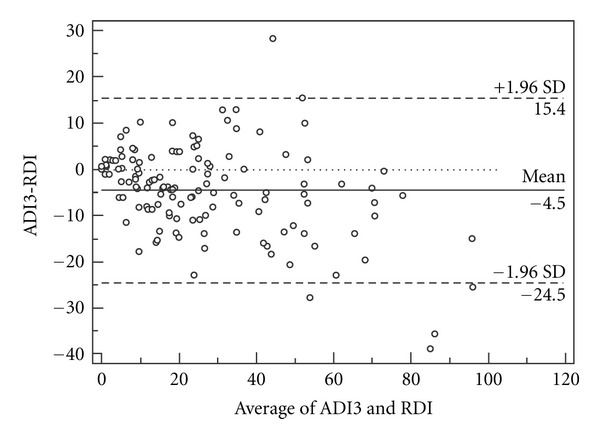
Bland-Altman plot of ADI3 and the respiratory disturbance index (RDI) from PSG (data storage rate 0.5 Hz).

**Figure 3:**
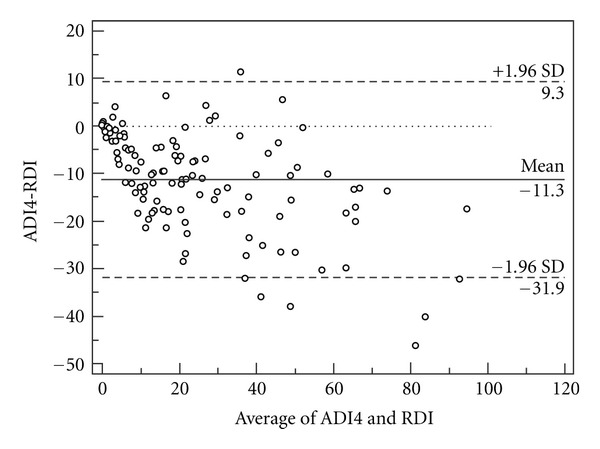
Bland-Altman plot of ADI4 and the respiratory disturbance index (RDI) from PSG (data storage rate 0.5 Hz).

The agreements between the ADI (data storage rate 0.5 Hz) and RDI are shown in Figures 1, 2, and 3. The mean difference between the ADI2, 3, and 4 and the RDI were 4.8 ± 11.5, −4.5 ± 10.2, and −11.3 ± 10.5, respectively. The intraclass correlation coefficients between the ADI and the RDI were ADI2: 0.86 (CI95% 0.77–0.91), ADI3: 0.89 (CI95% 0.81–0.93), and ADI4: 0.795 (CI95% 0.19–0.922).

### 3.3. ROC Curves Analysis, Sensitivity, Specificity, and Ratio Likelihood from Oximetry

The performance of the oximeters with different data storage rates is shown in Tables 2 and 3. There was no statistically significant difference in the accuracy of the oximeter with different data storage rates. 119 patients presented valid oximetry data with a sample rate of 0.5 Hz. The ADI cutoff points with the best sensitivity and specificity in these subjects are shown in Table 4.

### 3.4. False Negative and Positive Patient Characteristics

The lowest oximetry sensitivity was observed with a data storage rate of 0.5 Hz (criteria: ADI3 2 s > 10.4, OSAS = RDI ≥ 5). There were 16 false negative cases with this cutoff point. FN patients showed a lower BMI and RDI than true positive patients (TP). Also, they had a higher baseline SO_2_ and percentage of RERAs than TP patients (see Table 5). The lowest specificity was observed with a data storage rate of 0.25 Hz (criteria: ADI3 4 s > 9.1, OSAS = RDI ≥ 15) with 4 false positive cases. These patients had a lower baseline SO_2_ and a trend towards higher BMI than the true negative patients (SO_2_ 94 ± 1.4 versus SO_2_ 96.2 ± 1.2, *P* 0.04, BMI 29.2 ± 8.7 versus 25.1 ± 3.9, *P* 0.42). It was also noted that one subject had COPD and other a scoliosis of the thoracic spine.

## 4. Discussion

The main finding of this study was that the data storage rate of the oxygen saturation of WristOx 3100 did not affect its ability to diagnose subjects with suspected OSAS. Other authors have evaluated the influence of the data storage rate of the SO_2_ on the performance of the oximeter to quantity oxygen desaturation, but none has assessed the accuracy of pulse oximetry as a diagnostic test for OSAS using different data storage rates of SO_2_. Wiltshire et al. [11] studied 16 subjects in the sleep laboratory with PSG and two oximeters (Ohmeda Biox 3740) configured with data storage rates of 0.08 Hz and 0.5 Hz. Signal averaging defaulted to 6 s was used. The oxygen desaturation index of 4% (ODI4) on average was 3.2/h (range 0.1 to 18.3/h) for stored data every 12 s and 8.34/h (range 0.2 to 22.8/h) for SO_2_ saved every 2 s. The mean difference between both recording settings was −5.2 ± 4.4/h. Davila et al. [10] studied 75 patients with suspected OSAS by PSG and three oximeters (Ohmeda Biox 3740). These authors used three averaging times (3, 6, and 12 s) with online and memory displays of those data at each recording setting to calculate the index of desaturation. There was a significant effect on the memory mode of desaturation index, regardless of the averaging time of the SO_2_. The oxygen desaturation index of 3% (ODI3) was significantly higher with the memory display of 3 s than with 6 s and 12 s (14.8 ± 15.3/h, 8.9 ± 10.4/h, and 5.3 ± 6.6/h, *P* < 0.01). Using as a definition of a positive oximetry ODI3 ≥ 5, the percentages of negative oximetry for the three situations were 14% (3 s), 20% (6 s), and 28% (12 s). 

In contrast to these studies, we observed a negligible difference between the adjusted desaturation indexes (ADIs) with different data storage rates (see Table 6) that did not influence the diagnostic accuracy of oximetry even with the lower data storage rate (see Table 3). The most likely explanation for the discrepancy between our study and previous reports lies in the number of SO_2_ data stored in the memory of the oximeters. The minimum data storage rate of WristOx 3100 is higher than the Ohmeda 3740 (0.25 Hz versus 0.08 Hz, resp.). It suggests that saving data every 12 s is not sufficient to detect all episodes of oxygen desaturation in patients with OSAS, but collecting a value of SO_2_ every 4 s was sufficient to avoid a dropoff in resolution seen with lower rates data storage. Also, the algorithm used by the oximeters to average SO_2_, and distinct technologies could explain some of the differences observed. While the Ohmeda 3740 averaged a period of time (3, 6, or 12 s), the averaging of the WristOx 3100 is based on the number of heart beats, this allowing to adjust the SO_2_ for slower or faster heart rates. In addition, rather than a strictly linear averaging (i.e., mathematical mean or average), the WristOx 3100 uses an exponential averaging which allows the most recent data to be given more weight than the older data. Finally, although the recording conditions between the oximeters may be similar, distinct technologies could explain the differences observed in the number and depth of the oxygen desaturations [3–5].

**Figure 4:**
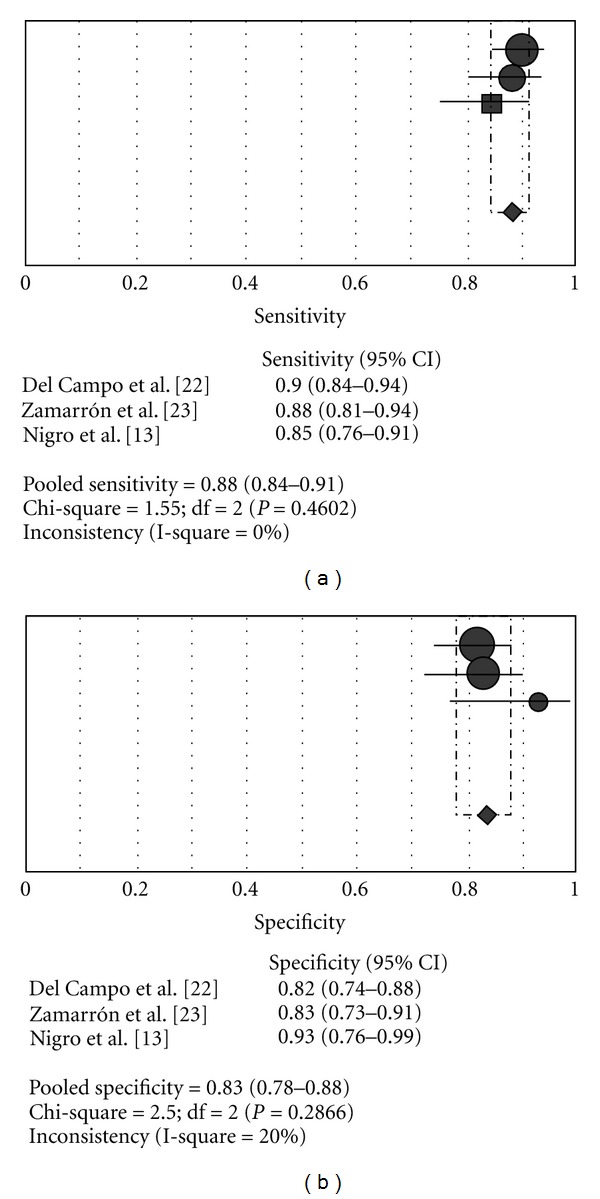
Oximetry versus polysomnography. OSAS criterion: AHI/RDI ≥ 10. Data storage rate oximeters: 0.2 Hz (Del Campo et al./Zamarron et al.), and 0.25 Hz (Nigro et al.).

**Figure 5:**
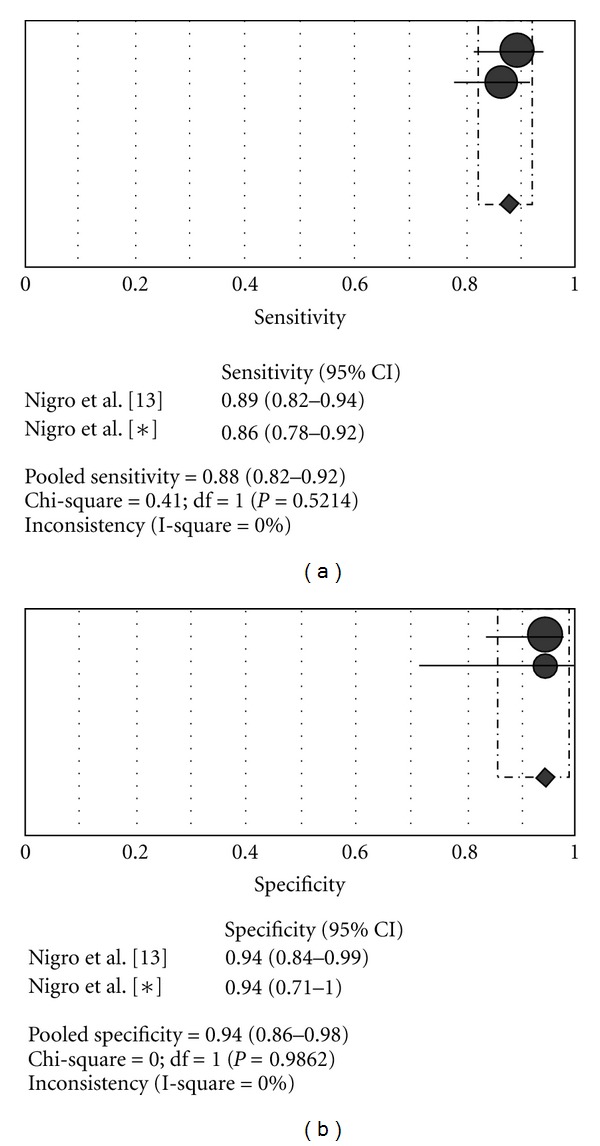
Oximetry versus polysomnography. OSAS criterion: AHI/RDI ≥ 5 (data storage rate oximeters: 0.5 Hz). (*): current study.

Clinical guidelines for the use of unattended portable monitors in the diagnosis of obstructive sleep apnea in adult patients recommend using an oximeter with a fast averaging time (≤3 s) and a high data storage rate for the assessment of oxygen saturation [21]. However, the recommendations have not mentioned the value of the data storage rate of SO_2_. Our observations suggest that a minimum data storage rate of 0.25 Hz would be sufficient to avoid loss of resolution oximetry to detect oxygen desaturation during sleep studies. In line with this hypothesis, two reports [22, 23] using a pulse oximeter with data storage rate of 0.2 Hz and mathematical analysis of the SO_2_ signal showed a sensitivity and specificity similar to the current study without heterogeneity between the studies (pooled sensitivity: 88%, 95% CI 84.4–91.2: pooled specificity: 83.4% 95% CI 78–88) (see Figure 4). In places where the access to polysomnography is difficult or when there is a long waiting list, the home oximetry, associated with a complete sleep history, is a useful tool as a first approach in patients with probable OSAS. In this context, clinicians and technicians must be aware of the influence of data storage rate on the performance of the oximetry. Thus, a minimum data storage rate of 0.25 Hz should be used to avoid losing resolution in the detection of oxygen desaturation associated with respiratory events. The re-evaluation of WristOx 3100 for subjects with suspected OSAS showed a performance similar to that previously reported, with a high sensitivity and specificity insubstantially changed by the 3 different data storage rates used (see Figure 5). Nevertheless, we cannot draw valid conclusions about the accuracy of the WristOx 3100 to detect or exclude OSAS outside the sleep laboratory without technical control even though it is likely that the performance of WristOx is similar at home since it has been reported that the accuracy of a level 4 device compared with PSG at home was similar to that observed in the sleep laboratory [24]. Also, the cutoff point hereby reported for the diagnosis of SAHS should be taken cautiously because OSAS prevalence in the general population is lower than in our study sample, which could reduce the diagnosis capacity of the WristOx 3100 oximeter. Finally, as no outcome measure was evaluated, we cannot know the clinical relevance of our findings for the initial management of OSAS-suspected patients.

In conclusion, the ability of WristOx 3100 to detect patients with OSAS was not affected by the data storage rate of the oxygen saturation signal. Both memory capacities of 0.25, 0.5, and 1 Hz, showed a similar performance for diagnosis of OSAS in our study population. According to these observations, the minimum data storage rate tested (0.25 Hz) has proved adequate for home oximetry with the WristOx 3100.

However, further studies are needed to confirm whether these findings can be applied to other models of oximeters.

## Figures and Tables

**Table 1:** Patient characteristics.

Patient number	101
Age (years)*	52.3 ± 16

Men (%)	77 (76.2)

BMI (body mass index-kg/m^2^)*	27.4 (24.9–31.1)

Prevalence of OSAS (%)	
(i) RDI ≥ 5	84 (83.2)
(ii) RDI ≥ 10	76 (75.2)
(iii) RDI ≥ 15	69 (68.3)

Patients without OSAS (RDI < 5) (%)	17 (16.8)

Severity of OSAS (%)	
(i) RDI ≥ 5–< 15	15 (17.9)
(ii) RDI ≥ 15–< 30	33 (39.3)
(iii) RDI ≥ 30	36 (42.9)

PSG	
(i) TRT (total recording time-min)**	422.7 (419.7–430.1)
(ii) TST (total sleep time-min)*	349.9 ± 40.2
(iii) TWT (total wakefulness time-min)**	61.5 (45.8–92.4)
(iv) SE (sleep efficiency)**	0.85 (0.78–0.89)
(v) TNREM (min)*	294.2 ± 34.2
(vi) TREM (min)*	55.2 ± 24.5
(vii) AHI (apnea/hypopnea index)**	20.7 (7.6–37.6)
(viii) AI (apnea index)**	2.4 (0.62–12.7)
(ix) HI (hypopnea index)**	14.7 (4.9–23.4)
(x) RDI (respiratory disturbance index)**	22.6 (10.2–39.9)

Comorbidities (%)	
(i) Hypertension	40 (39)
(ii) Coronary heart disease	7 (6.9)
(iii) Cerebrovascular ischemia	3 (2.9)
(iv) Cardiac arrhythmia	14 (13.9)
(v) Asthma	2 (2)
(vi) COPD	7 (6.9)
(vii) Rhinitis	40 (39)
(viii) Diabetes	11 (10.9)
(ix) Hypothyroidism	11 (10.9)

Data are presented as mean ± SD*, median (25–75% percentiles) **, or *n* (%). OSAS: obstructive sleep apnea syndrome.

TNREM: total stages 1+2+3+4; TREM: total amount of REM sleep.

**Table 2:** Area under the ROC curves of the adjusted desaturation index with different data storage rates.

	AUC-ROCs (CI95%)		
	Data storage rates WristOx 3100		
	1 Hz	0.5 Hz	0.25 Hz
OSAS = RDI ≥ 5 Oximetry criteria			
ADI2	0.965 (0.909–0.992)	0.948 (0.885–0.982)	0.958 (0.898–0.988)*
ADI3	0.966 (0.91–0.992)	0.95 (0.888–0.984)	0.961 (0.903–0.989)*
ADI4	0.963 (0.905–0.990)	0.949 (0.887–0.983)	0.957 (0.897–0.987)*

OSAS = RDI ≥ 10 Oximetry criteria			
ADI2	0.947 (0.884–0.982)	0.945 (0.881–0.981)	0.947 (0.884–0.982)*
ADI3	0.948 (0.885–0.982)	0.950 (0.888–0.984)	0.951 (0.889–0.984)*
ADI4	0.954 (0.893–0.986)	0.946 (0.883–0.981)	0.942 (0.877–0.979)*

OSAS = RDI ≥ 15 Oximetry criteria			
ADI2	0.954 (0.893–0.986)	0.949 (0.887–0.983)	0.946 (0.882–0.981)*
ADI3	0.954 (0.883–0.982)	0.947 (0.883–0.982)	0.947 (0.884–0.982)*
ADI4	0.954 (0.894–0.986)	0.942 (0.877–0.979)	0.94 (0.875–0.978)*

RDI: respiratory disturbance index; AUC-ROC: area under the ROC curve; ADI2, 3, and 4: adjusted desaturations index ≥2%, 3%, and 4%; CI95%: confidence interval 95%; *P > 0.05 between ADI 1 Hz, 0.5 Hz and 0.25 Hz.

**Table 3:** Sensitivity and specificity of adjusted desaturation index with different data storage rates.

Data storage rates WristOx 3100								
	1 Hz			0.5 Hz			0.25 Hz	
	Sensitivity (CI95%)	Specificity (CI95%)		Sensitivity (CI95%)	Specificity (CI95%)		Sensitivity (CI95%)	Specificity (CI95%)
OSAS = RDI ≥ 5 Oximetry criteria			OSAS = RDI ≥ 5 Oximetry criteria			OSAS = RDI ≥ 5 Oximetry criteria		
ADI2 > 19.7	85.7 (76.4–92.4)	100 (80.5–100)	ADI2 > 19.5	85.7 (76.4–92.4)	94.1 (71.3–99.9)	ADI2 > 18.4	85.7 (76.4–92.4)	100 (80.5–100)
ADI3 > 7.6	89.3 (80.6–95)	94.1 (71.3–99.9)	ADI3 > 10.4	81 (70.9–88.7)	100 (80.5–100)	ADI3 > 7.3	86.9 (77.8–93.3)	100 (80.5–100)
ADI4 > 2.1	94 (86.7–98)	88.2 (63.6–98.5)	ADI4 > 3.7	88.1 (79.2–94.1)	94.1 (71.3–99.9)	ADI4 > 3.6	89.3 (80.6–95)	100 (80.5–100)

OSAS = RDI ≥ 10 Oximetry criteria			OSAS = RDI ≥ 10 Oximetry criteria			OSAS = RDI ≥ 10 Oximetry criteria		
ADI2 > 19.7	92.1 (83.6–97)	92 (74–99)	ADI2 > 23.3	84.2 (74–91.6)	96 (79.6–99.9)	ADI2 > 18.4	90.8 (81.9–96.2)	88 (68.8–97.5)
ADI3 > 9.9	89.5 (80.3–95.3)	92 (74–99)	ADI3 > 10.6	86.8 (77.1–93.5)	96 (79.6–99.9)	ADI3 > 8.6	89.5 (80.3–95.3)	92 (74–99)
ADI4 > 5.7	85.5 (75.6–92.5)	96 (79.6–99.9)	ADI4 > 5.6	82.9 (72.5–90.6)	100 (86.3–100)	ADI4 > 4	88.2 (78.7–94.4)	92 (74–99)

OSAS = RDI ≥ 15 Oximetry criteria			OSAS = RDI ≥ 15 Oximetry criteria			OSAS = RDI ≥ 15 Oximetry criteria		
ADI2 > 22.8	94.2 (85.8–98.4)	90.6 (75–98)	ADI2 > 23.3	89.9 (80.2–95.8)	90.6 (75–98)	ADI2 > 22.1	89.9 (80.2–95.8)	87.5 (71–96.5)
ADI3 > 12.3	94.2 (85.8–98.4)	90.6 (75–98)	ADI3 > 10.8	91.3 (82–96.7)	90.6 (75–98)	ADI3 > 9.1	92.7 (83.9–97.6)	87.5 (71–96.5)
ADI4 > 5.8	91.3 (82–96.7)	93.7 (79.2–99.2)	ADI4 > 6.1	87 (76.7–93.9)	96.9 (83.8–99.9)	ADI4 > 6.8	84.1 (73.3–91.8)	96.9 (83.8–99.9)

RDI: respiratory disturbance index; ADI2, 3, and 4: adjusted desaturations index ≥2%, 3%, and 4%; LR (+) and (−): positive and negative likelihood ratio; CI95%: confidence interval 95%.

**Table 4:** Sensitivity and specificity of adjusted desaturation index with a data storage rate of 0.5 Hz.

	Sensitivity (CI95%)	Specificity (CI95%)	LR + (CI95%)	LR − (CI95%)
OSAS = RDI ≥ 5 oximetry criteria				
ADI2 > 19.5	86.3 (78–92.3)	94.1 (71.3–99.9)	14.7 (12.7–16.9)	0.15 (0.02–1)
ADI3 > 10.4	80.4 (71.4–87.6)	100 (80.5–100)	∞	0.20
ADI4 > 3.7	85.3 (76.9–91.5)	94.1 (71.3–99.9)	14.5 (12.6–16.7)	0.16 (0.02–1.1)

OSAS = RDI ≥ 10 oximetry criteria				
ADI2 > 23.3	84.6 (75.5–91.3)	92.9 (76.5–99.1)	11.8 (10.4–13.6)	0.17 (0.04–0.7)
ADI3 > 10.6	86.8 (78.1–93)	96.4 (81.7–99.9)	24.3 (21.8–27.1)	0.14 (0.02–1)
ADI4 > 5.6	82.4 (73–89.6)	100 (87.7–100)	∞	0.18

OSAS = RDI ≥ 15 oximetry criteria				
ADI2 > 23.3	90.4 (81.9–95.7)	88.9 (73.9–96.9)	8.1 (7.1–9.3)	0.11 (0.03–0.3)
ADI3 > 10.8	90.4 (81.9–95.7)	91.7 (77.5–98.2)	10.9 (9.6–12.2)	0.11 (0.03–0.4)
ADI4 > 6.1	86.7 (77.5–93.2)	97.2 (85.5–99.9)	31.2 (28.2–34.5)	0.14 (0.02–1)

RDI: respiratory disturbance index; ADI2, 3, and 4: adjusted desaturations index ≥2%, 3%, and 4%; LR (+) and (−): positive and negative likelihood ratio; CI95%: confidence interval 95%.

**Table 5:** False negative cases.

	False negative	True positive	*P*
Number	16	69	
BMI (kg/m^2^)*	25.7 ± 3.6	30.7 ± 6	<0.01
Baseline SO_2 _*	96 ± 1.36	94 ± 1.63	<0.01
RDI*	12.8 ± 5.4	37.6 ± 20.2	<0.01
% hypopneas	62.5	62	0.1
% RERAs	23.4	7	0.01

Values are expressed as mean ± SD*.

**Table 6:** Adjusted desaturation index with different data storage rates.

	1 Hz	0.5 Hz	0.25 Hz
ADI2	33.9 (2)	33.4 (1.95)	33.5 (2.1)
ADI3	24.9 (2)*	24 (1.96)*	23.8 (2)**
ADI4	18.2 (1.8)*	17.4 (1.74)*	17.3 (1.74)**

Values are expressed as mean (std. error). ADI2, 3, and 4: adjusted desaturations index ≥2%, 3%, and 4%.

*Pairwise comparisons between ADI3/4 1 s and ADI3/4 2 s: *P* 0.01.

**Pairwise comparisons between ADI3/4 1 s and ADI3/4 4 s: *P* 0.001.

## References

[1] Shepard JW, Kryger MH, Roth T, Dement WC (1994). Cardiorespiratory changes in obstructive sleep apnea. *Principles and Practice of Sleep Medicine*.

[2] Nigro CA, Rhodius EE (2005). Variation in the duration of arousal in obstructive sleep apnea. *Medical Science Monitor*.

[3] Brouillette RT, Lavergne J, Leimanis A, Nixon GM, Ladan S, McGregor CD (2002). Differences in pulse oximetry technology can affect detection of sleep-disorderd breathing in children. *Anesthesia and Analgesia*.

[4] Hannhart B, Haberer JP, Saunier C, Laxenaire MC (1991). Accuracy and precision of fourteen pulse oximeters. *European Respiratory Journal*.

[5] Zafar S, Ayappa I, Norman RG, Krieger AC, Walsleben JA, Rapoport DM (2005). Choice of oximeter affects apnea-hypopnea index. *Chest*.

[6] West P, George CF, Kryger MH (1987). Dynamic in vivo response characteristics of three oximeters: Hewlett-Packard 47201A, Biox III, and Nellcor N-100. *Sleep*.

[7] Farré R, Montserrat JM, Ballester E, Hernández L, Rotger M, Navajas D (1998). Importance of the pulse oximeter averaging time when measuring oxygen desaturation in sleep apnea. *Sleep*.

[8] Kendrick AH, Wiltshire N, Catterall JR (1996). Effect of signal averaging time (Tsa) on on-line pulse oximetry used for overnight sleep recordings. *American Journal of Respiratory and Critical Care Medicine*.

[9] Davila D, Richards K, Marshall B (1999). Oximeter’s acquisition settings influence its performance and clinical decision making. *Sleep*.

[10] Davila DG, Richards KC, Marshall BL (2002). Oximeter performance: the influence of acquisition parameters. *Chest*.

[11] Wiltshire N, Kendrick AH, Catterall JR (2001). Home oximetry studies for diagnosis of sleep apnea/hypopnea syndrome: limitation of memory storage capabilities. *Chest*.

[12] Davila DG, Richards KC, Marshall BL (2003). Oximeter’s acquisition parameter influences the profile of respiratory disturbances. *Sleep*.

[13] Nigro CA, Aimaretti S, Gonzalez S, Rhodius E (2009). Validation of the WristOx 3100*™* oximeter for the diagnosis of sleep apnea/hypopnea syndrome. *Sleep and Breathing*.

[14] Netzer NC, Stoohs RA, Netzer CM, Clark K, Strohl KP (1999). Using the Berlin Questionnaire to identify patients at risk for the sleep apnea syndrome. *Annals of Internal Medicine*.

[15] Rechtschafen A, Kales A (1968). *A Manual of Standarized Technology, Techniques and Scoring System for Sleep Stages of Human Subjects*.

[16] Bonnet M, Carley D, Carskadon M (1992). EEG arousals: scoring rules and examples. A preliminary report from the Sleep Disorders Atlas Task Force of the American Sleep Disorder Association. *Sleep*.

[17] (2005). Consenso Nacional sobre el Síndrome Apneas-Hipopneas del Sueño del Grupo Español de Sueño Definición y concepto, fisiopatología, clínica y exploración del SAHS. *Archivos de Bronconeumologia*.

[18] Ayappa I, Norman RG, Krieger AC, Rosen A, O’Malley RL, Rapoport DM (2000). Non-invasive detection of respiratory effort-related arousals (RERAs) by a nasal cannula/pressure transducer system. *Sleep*.

[19] Sala H, Nigro C, Rabec C, Guardia AS, Smurra M (2001). Consenso Argentino de Trastornos Respiratorios Vinculados al Sueño. *Medicina*.

[20] Guilleminault C, Bassiri A, Kryger MH, Roth T, Dement WC (2005). Clinical features and evaluation of obstructive sleep apnea-hypopnea syndrome and upper airway resistance syndrome. *Principles and Practice of Sleep Medicine*.

[21] Collop NA, Anderson WM, Boehlecke B (2007). Clinical guidelines for the use of unattended portable monitors in the diagnosis of obstructive sleep apnea in adult patients. *Journal of Clinical Sleep Medicine*.

[22] del Campo F, Hornero R, Zamarrón C, Abasolo DE, Álvarez D (2006). Oxygen saturation regularity analysis in the diagnosis of obstructive sleep apnea. *Artificial Intelligence in Medicine*.

[23] Zamarrón C, Gude F, Barcala J, Rodriguez JR, Romero PV (2003). Utility of oxygen saturation and heart rate spectral analysis obtained from pulse oximetric recordings in the diagnosis of sleep apnea syndrome. *Chest*.

[24] Zou D, Grote L, Peker Y, Lindblad U, Hedner J (2006). Validation a portable monitoring device for sleep apnea diagnosis in a population based cohort using synchronized home polysomnography. *Sleep*.

